# Lagos lagoon sediment organic extracts and polycyclic aromatic hydrocarbons induce embryotoxic, teratogenic and genotoxic effects in *Danio rerio* (zebrafish) embryos

**DOI:** 10.1007/s11356-016-6490-y

**Published:** 2016-04-11

**Authors:** Temitope O. Sogbanmu, Eszter Nagy, David H. Phillips, Volker M. Arlt, Adebayo A. Otitoloju, Nic R. Bury

**Affiliations:** Ecotoxicology and Conservation Unit, Department of Zoology, Faculty of Science, University of Lagos, Akoka, 101017 Lagos Nigeria; Division of Analytical and Environmental Sciences, Faculty of Life Sciences and Medicine, MRC-PHE Centre for Environment and Health, King’s College London, Franklin-Wilkins Building, 150 Stamford Street, London, SE1 9NH UK; Division of Diabetes and Nutritional Sciences, Faculty of Life Sciences and Medicine, King’s College London, Franklin-Wilkins Building, 150 Stamford Street, London, SE1 9NH UK

**Keywords:** Sediment, Teratogenicity, Embryotoxicity, Genotoxicity, Lagos lagoon, Zebrafish embryos

## Abstract

An expansion of anthropogenic activity around Lagos lagoon, Nigeria, has raised concerns over increasing contaminants entering the lagoon’s ecosystem. The embryotoxicity, teratogenicity and genotoxicity of sediment organic extracts from four sampling zones around Lagos lagoon, Ilaje, Iddo, Atlas Cove and Apapa, as well as the dominant polycyclic aromatic hydrocarbons (PAHs) identified in water measured during the wet season (naphthalene, phenanthrene, pyrene, benzo[a]pyrene and a mixture of these), were assessed with *Danio rerio* embryos. Embryos were exposed to varying concentrations of toxicants from 0–72 h post-fertilization (hpf). Embryotoxicity at 72 hpf showed a dose-dependent increase in mortality upon exposure to extracts from all zones, except Atlas Cove. Similarly, higher levels of teratogenic effects, such as increased oedema, and haemorrhage and developmental abnormalities resulted from exposure to extracts from Ilaje, Iddo and Apapa zones. Treatment with single PAHs revealed that significant levels of detrimental effects were obtained only for phenanthrene. The modified comet assay revealed that the oxidative damage to DNA was generally low (<12 %) overall for all sediment extracts, but was significantly elevated with Ilaje and Iddo sediment extracts when compared with solvent controls. Oxidative damage was observed with the single PAHs, phenanthrene and benzo[a]pyrene, as well as with the PAH mixture. This study highlights that Lagos lagoon sediment extracts have teratogenic, embryotoxic and genotoxic properties, which are likely due to the high molecular weight PAHs present in the extracts, some of which are known or are suspected human carcinogens.

## Introduction

Lagoons are ecologically and economically important aquatic ecosystems that provide water and food, primarily in the form of fish, to many people worldwide. For example, the Lagos lagoon in Nigeria provides a number of important ecosystem services that include fish supply for the indigenous fishing communities of Ilajes and Ijaws (Ajagbe et al. 2012). It is a part of the continuous system of lagoons and creeks that are found along the coast of Nigeria from the border with the Republic of Benin to the Niger Delta. The major outlet of freshwater is at Lagos, Nigeria, where it forms an extensive harbour (Okoye et al. 2010). Within the lagoon, the tidal range is between 0.3 and 1.3 m and it is generally relatively shallow between 0.5 and 2 m and deep in most parts with a maximum of about 5 m. However, to accommodate shipping activity the harbour is routinely dredged to a depth of 25 m.

An increase in the discharge of domestic, municipal and industrial effluents, as well as contaminants associated with sand mining and shipping activities, threatens the ecosystem services that Lagos lagoon provides (Balogun et al. 2011; Amaeze et al. 2012; Alani et al. 2012). For example, the anthropogenic activities in and around Lagos lagoon have been suggested as the major sources of polycyclic aromatic hydrocarbons (PAHs) (Alani et al. 2012) contributing to the significant decline of fishery resources and threatening their long-term sustainability. Singh et al. (1995) reported a reduction in annual fish production by over fivefold between 1970 and 1990, and Amaeze et al. (2012) recently reported significant declines in fish abundance and diversity.

The sediments within the lagoon range between mud, sandy mud, muddy sand and sand (Ajao and Fagade 1990) and are considered a reservoir or sink for pollutants especially hydrophobic organic contaminants, which can be resuspended in the water column by natural and/or anthropogenic phenomena (e.g. tides, dredging and flooding) (Wölz et al. 2009; Lesueur et al. 2015). Hydrophobic organic contaminants in sediments are routinely identified or quantified, but this is often inadequate for assessing the toxic potential of the sediment extracts to living organisms because of the possible additive, synergistic or antagonistic interactions between components of the complex mixture of compounds present (Amaeze et al. 2015). In contrast, the use of bioassays can reflect the combined toxic effects of all the contaminants present in the samples and, if coupled with chemical analysis, could offer more reliable information on the risk of chemical contamination (Long et al. 1995; Yang et al. 2010).

The assessment of DNA alterations in aquatic organisms has been shown to be a highly suitable method for evaluating the genotoxic effects from environmental contaminants (Jha 2008; Frenzilli et al. 2009; Al-Subiai et al. 2012), and because it is a measure of DNA damage, it has implication not just for individuals but also for populations (Jha 2008). The single cell gel electrophoresis (SCGE) or comet assay is a relatively quick and reliable method to detect DNA damage such as single- and double-strand breaks, alkali-labile lesions and apurinic sites (Tice et al. 2000). One of its major advantages is that DNA strand breaks form quickly following exposure to genotoxicants, allowing for an early response evaluation of genotoxic effects (Frenzilli et al. 2009). The assay can be modified by the addition of endonucleases to assess oxidative damage to DNA (Azqueta and Collins 2013). For example, formamidopyrimidine DNA glycosylase (FPG) removes oxidised purines (Albertini et al. 2000), but alkylated DNA lesions may also be detected by this enzyme (Azqueta and Collins 2013). The comet assay is not restricted for use in genotoxic hazard classification of chemicals but can also be used in determining the genotoxicity of complex environmental matrices such as sewage treatment plant effluents (Llorente et al. 2012), marine coastal sediments (Davoren et al. 2005; Šrut et al. 2011; Amaeze et al. 2015) and sediments from a gypsum mining area (Ternjej et al. 2013) and the River Danube (Boettcher et al. 2010). The majority of these studies have used cell lines for their analysis of DNA damage, but the comet assay has been adapted for embryos of zebrafish, *Danio rerio*, and used to assess sediment extracts from the Laguna lake in the Philippines (Kosmehl et al. 2008).

Ajagbe et al. (2012) recommended the need for more studies to establish the state of pollution in the Lagos lagoon and the levels of pollutants that are detrimental to the ecosystem health and to humans due to the consumption of contaminated fish. Currently, there is a dearth of information on the embryotoxic, teratogenic and genotoxic effects of the organic fractions of sediments on aquatic life in Nigeria. Only a few relevant methods have been developed to assess sediment toxicity and its ecological impacts in a cost-effective way. Fish model species such as zebrafish, because of their transparent early embryo and well-characterized developmental stages, are emerging as reliable test organisms for toxicity testing (Strahle et al. 2012). Recent international guidelines have approved the use of early life stages of fish to document acute and sub-lethal effects of pollutants (OECD 2013), including those present in sediments (Kosmehl et al. 2007, 2008). Consequently, this study aimed to use *D. rerio* embryos as a fish model to assess the effects of organic solvent extracts of sediments and selected PAHs known to be present in the sediments to assess the embryotoxic (mortality) and teratogenic effects (hatch rate, heartbeat rate and developmental effects) and induced genotoxicity via a modified comet assay (i.e. oxidative damage to DNA).

## Materials and methods

### Chemicals and suppliers

Chemicals were purchased from the following sources:

Dichloromethane (DCM), analytical reagent grade 99.99 %, hexane, laboratory reagent grade, and acetone, analytical reagent grade 99.99 %, were all from Fisher Scientific, Loughborough, UK. Dimethylsulphoxide (DMSO), molecular biology grade ≥99.9 %, benzo[a]pyrene (>96 % purity; CAS: 50-32-8 601-032-00-3), pyrene (99 % purity, CAS 129-00-0) and Pronase E (from *Streptomyces griseus*) were from Sigma-Aldrich, Gillingham, Dorset, UK. Naphthalene (99 % purity, CAS 91-20-3) and phenanthrene (98 % purity, CAS 85-01-8) were from Alfa Aesar, Ward Hill, MA, USA. FPG was kindly provided by Professor Andrew Collins from Oslo University, Norway. Ultrapure low melting point agarose (LMPA) and foetal bovine serum (FBS) were both from Life Technologies, Paisley, UK.

### Study location and sediment collection

**Fig. 1:**
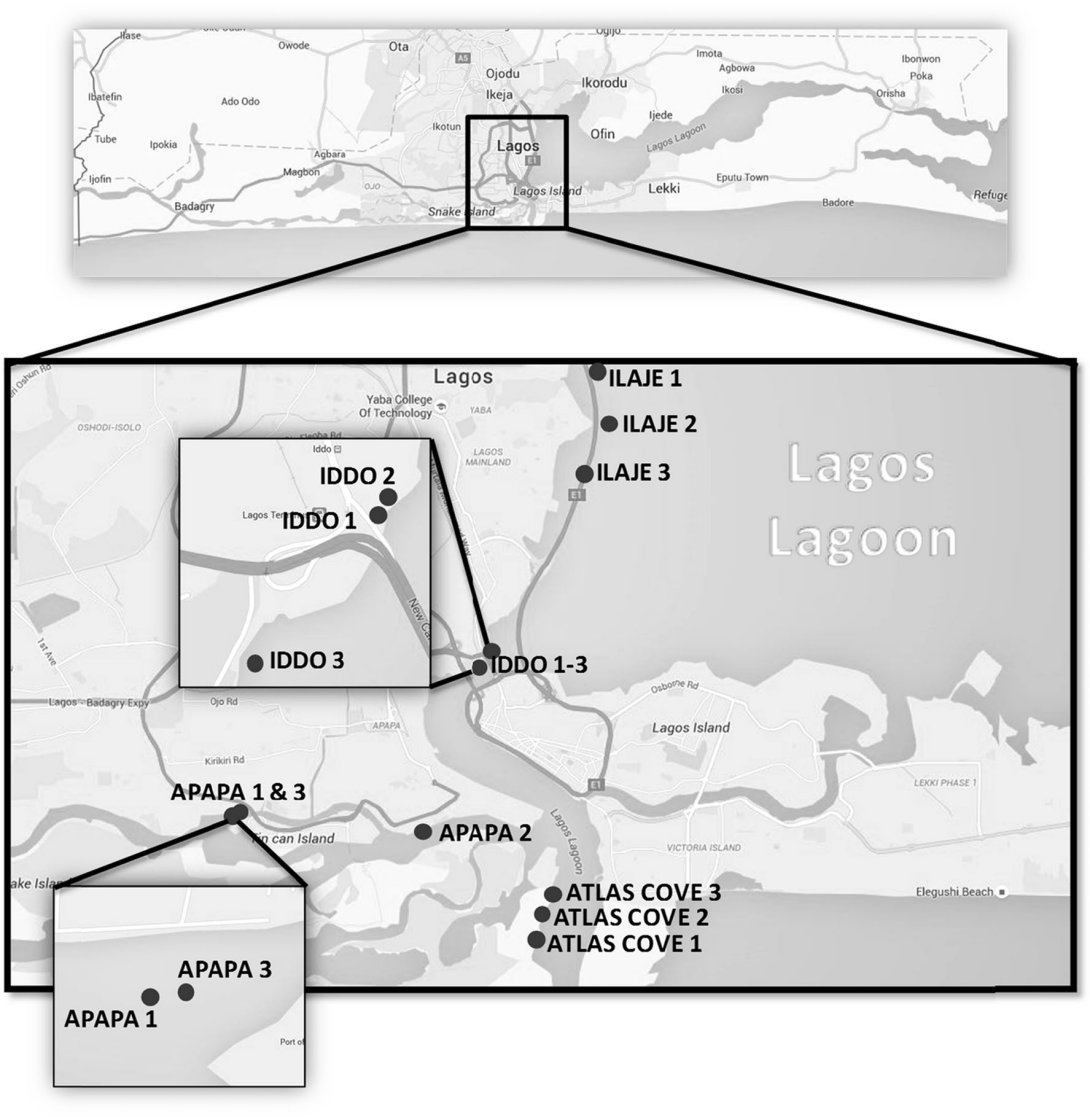
Map of Lagos lagoon showing sampling sites for sediment collection

Four sampling zones (Fig. 1) were selected based on the degree of anthropogenic activities in the areas along Lagos lagoon (see information in the previous study of Amaeze et al. 2015). Sampling was conducted in January 2014 (dry season), when surface sediment samples were collected with a stainless steel Ekman bottom sampler, air dried, wrapped with aluminium foil and stored at −20 °C prior to transportation to King’s College London, UK. Samples were collected as composites of three sampling stations per zone (Fig. 1).

### Sediment PAH measurements

Sediments were dried and ground in a mortar. To 20 g of sample, 100 mL hexane/DCM (3:1) was added, and the solution was sonicated for 2 h. The organic layer was filtered through a funnel containing anhydrous sodium sulphate and dried through evaporation over a stream of nitrogen. To separate the aliphatic and aromatic hydrocarbons, the sediment extract was loaded onto a 10-mL glass column packed with activated alumina pre-cleaned with hexane. The aliphatic compounds were eluted with 20 mL of hexane, aromatic fractions with 20 mL hexane/DCM (3:1), and the most polar compounds were removed with 20 mL DCM alone. The combined extracts were concentrated to 1 mL over a stream of nitrogen before gas chromatography (GC) analysis using Hewlett Packard Gas Chromatograph 6890 with flame ionization detector and HP ChemStation Rev. A 09.01 [1206] software.

A total of 16 PAHs (naphthalene, acenaphthylene, acenaphthene, fluorene, phenanthrene, anthracene, fluoranthene, pyrene, benz[a]anthracene, chrysene, benzo[b]fluoranthene, benzo[k]fluoranathene, benzo[a]pyrene, indeno[1,2,3-cd]pyrene, dibenz[a,h]anthracene and benzo[ghi]perylene) were analysed using modified methods of ASTM D3328-06 (2013) and ASTM D3415-98 (2011). PAH analysis was conducted with the following GC conditions: injection temperature at 250 °C, flame ionization detector (FID) at 320 °C, and separation on a non-polar, general-purpose and industry-standard capillary column HP-1 with length 30 m and ID 0.25 μm. The temperature gradient programme started with an initial temperature of 60 °C, upon which the first rate increased with 15 °C/min for 14 min maintained for 3 min, followed by a second rate of 10 °C/min for 5 min maintained for 4 min. The mobile phase (carrier gas) was nitrogen.

### Sediment organic contaminant extraction for in vivo studies

Sediment organic extraction was conducted as previously described with slight modifications (Schnell et al. 2013; Amaeze et al. 2015). Upon arrival at King’s College London, sediments samples were freeze dried. Sediments were ground in a clean ceramic crucible and passed through a 63-μm sieve. Subsequently, 2.5 g was weighed into glass vials and, with the addition of 10 mL of DCM/hexane (1:1), samples were sonicated in a water bath sonicator (Decon F5200b, Patterson Scientific) for 10 min at 4 °C and then centrifuged at 2000×*g* for 10 min at 4 °C (Eppendorf Centrifuge 5810R). The supernatant was transferred into a clean glass vial. Sediments were further extracted using DCM/acetone (1:1), and the process of sonication and centrifugation was repeated as above. Supernatants were pooled and reduced to 5 mL over a stream of nitrogen. Fifty milligrams of activated copper was added to each extract and stored overnight at 4 °C. The next day, the supernatant was carefully aspirated, transferred into new glass vials and evaporated over a stream of nitrogen to complete dryness. Extracts were then reconstituted with 250 μL of DMSO and stored at −20 °C until use. The stock sediment organic extract solution was equivalent to 10 g dry weight sediment equivalent extract (eQsed) per millilitre.

### Zebrafish embryo exposures to sediment extracts and PAHs

Wild-type adult *D. rerio*, strain AB, were obtained from the breeding colony at King’s College London. The fish were maintained in a recirculating system at pH 7–8, temperature 26–29 °C and light/dark period of 12:12 h. The fish were kept at a ratio of 2:1 female/male with a total of nine fish per tank, and embryos were collected from three tanks. Fish were fed daily (morning and evening) with commercially available artificial zebrafish diet supplemented with brine shrimp + omega-3 (Tropical Marine Centre, Hertfordshire, UK). Spawned eggs and embryos were collected approximately 1 h after daylight commenced. Embryos were collected and washed at least three times with embryo medium/ISO standardized water (117.6 mg/L CaCl_2_·2H_2_O, 49.3 mg/L MgSO_4_·7H_2_O, 25.9 mg/L NaHCO_3_, 2.3 mg/L KCl according to Kumar et al. 2013), and any dead/unfertilized eggs were discarded.

A total of 30 embryos (10 embryos in triplicates per concentration) were exposed up to 72 h post-fertilization (hpf) in 4 mL of ISO standardized water containing sediment organic extract concentrations (2.5, 6.25, 12.5 and 25 mg eQsed/mL) from each site. In addition, the embryos were incubated with varying concentrations (2.5, 25 and 50 μM) of naphthalene, phenanthrene, pyrene or benzo[a]pyrene on their own or as a mixture. The mixture contained the four PAHs in a ratio of 12:1:3:1 (naphthalene/phenanthrene/pyrene/benzo[a]pyrene) at levels designated low (L), medium (M) and high (H); see Table 1 for concentrations of individual PAHs in these mixtures. This ratio is based on the measured concentrations of the four PAHs in water from Lagos lagoon during the wet season (Sogbanum and Otitoloju, unpublished data). The embryos were incubated in a laboratory incubator (Innova 4200, New Brunswick Scientific, Edison, USA) at 29 ± 0.5 °C. Two controls were also included: embryo media alone and embryo media containing 0.25 % DMSO, with the maximum amount used as a vehicle for the extracts.

**Table 1:** Concentration of PAH mixtures presented as low (L), medium (M) and high (H) in the rations of 12:1:3:1 for naphthalene, phenanthrene, pyrene and benzo[a]pyrene

Compounds	Ratio	Low (L)	Medium (M)	High (H)
		μg*/*L (μM)		
Naphthalene	12	226.2 (1.68)	2262.4 (16.8)	4524.8 (33.6)
Phenanthrene	1	26.2 (0.14)	262.1 (1.4)	524.2 (2.8)
Pyrene	3	89.2 (0.44)	891.9 (4.4)	1783.8 (8.8)
Benzo[a]pyrene	1	37.6 (0.14)	370.6 (1.4)	741.1 (2.8)

### Embryotoxicity and teratogenicity

Embryotoxicity (mortality) and teratogenicity (developmental abnormalities, hatching and number of heart beats per minute) were assessed at 24, 48 and 72 hpf, with results at 72 hpf reported. Visual criteria to describe teratogenicity are taken from Kumar et al. (2013) (Table 2).

**Table 2:** Recorded abnormal developmental endpoints at 24, 48 and 72 hpf in zebrafish embryos

Endpoints	Description
*Lethal*	
Coagulation	Embryo coagulated with no structures
Heartbeats	Embryo has no visible heartbeat
*Teratogenic/developmental*	
Tail development	Tail is shorter than normal or curved and/or tail tip is malformed
Oedema	Oedema (swelling caused by fluid retention) is present in the yolk sac, pericardial region or both regions
Heart rate	Alterations to number of heart beats per minute (NHBpM)
*Cardiac morphology*	
Haemorrhage	Visualized as a pool of blood in a tissue or organ
Thrombosis	Observed as a stagnant blood flow or blood clot in the cardinal vein
Scoliosis	Abnormal curvature of the spine to the side

Adapted from Kumar et al. (2013)

### Genotoxicity assay–comet assay

After 24, 48 and 72 hpf, zebrafish embryos from the lowest and highest sediment concentration treatments (2.5 and 25 mg eQsed/ mL), and the various PAH treatments, along with controls, were placed in 0.5-mL microcentrifuge tubes containing 50 μL of 10 mg/mL Pronase E in phosphate-buffered saline (PBS) (Oxoid Ltd, Basingstoke Hampshire, England). After 5 min, the Pronase E and dissociated chorions were aspirated. The embryos were rinsed thrice with 200 μL pure PBS, then resuspended in 200 μL 10 % FBS in PBS and passed up and down a 200-μL pipette tip 20–25 times. The suspension was filtered through a 40-μm cell strainer (BD Falcon, Scientific Laboratory Supplies, Nottingham, UK) into a 50-mL conical centrifuge tube containing 2 mL of 10 % FBS in PBS. The resulting cell suspension was centrifuged for 10 min at 4 °C, 250×*g*, after which the supernatant was removed and cells washed in 1 mL pure PBS. This was further centrifuged at 200×*g* at 4 °C, for 8 min, supernatant aspirated and cells resuspended in 200 μL of PBS and stored on ice.

The comet assay was conducted under alkaline conditions according to Singh et al. (1988) with some modifications as follows. A minimum of 24 h prior to the comet assay, three-window PTFE diagnostic microscope slides (Thermo Scientific, Portsmouth) were pre-coated with 15 μL 0.75 % LMPA in PBS. After drying at 37 °C for 30 min, a second layer of 15 μL 0.75 % LMPA was applied onto the slides and left to dry overnight at 37 °C. All buffers used in the assay were pre-cooled, and samples were kept on ice. Twenty microliters of cell suspension was transferred into 200 μL of warm (37 °C exactly) LMPA and mixed by pipetting. From this, 30 μL was transferred onto each slide window and placed on a cold surface for 2–3 min until the gels solidified.

The slides were then placed in cold (4 °C) lysis buffer (0.25 M NaCl, 1 mM Tris-base, 10 mM EDTA, 0.1 %Triton X-100 at pH 10) for a minimum of 1 h. Following lysis, the slides were conditioned for 2 × 7.5 min in enzyme buffer (0.1 M KCl, 40 mM Hepes, 0.5 mM EDTA, 0.2 mg/mL FBS). Thereafter, 30 μL of enzyme buffer only (unmodified) or 30 μL of the FPG enzyme (54 ng/μL enzyme buffer) (modified) was pipetted onto each slide window. The slides were carefully covered with parafilm to retain moisture and incubated at 37 °C for 30 min.

After enzyme treatment, the slides were placed in cold (4 °C) alkaline/electrophoresis buffer (0.3 M NaOH, 1 mM EDTA) for 40 min, followed by electrophoresis in the same buffer for 20 min at 20 V, and subsequently placed in neutralization buffer (400 mM Tris-base, pH 7.4) for 5 min, distilled water for 5 min and fixed in absolute ethanol for 5 min. Lastly, slides were left to dry for at least 30 min before staining with 10 μg/mL ethidium bromide for imaging.

All slides were examined at ×40 magnification using a fluorescent microscope (Leica Microscope, Microsystems Wetzler GmbH, Germany) and equipped with a CCD camera (Marlin imaging device) and analysed using the Comet IV software (2006) (Perceptive Instruments, UK).

### Statistical analysis

For the embryotoxicity (mortality) and teratogenicity (hatching, developmental abnormalities and heart beat/rate) assays, results are presented as the average ± SE of triplicate wells (10 embryos per well) of three independent experiments and expressed as a percentage. For each endpoint, significant difference (*p* < 0.05) between the means were calculated on log-transformed data using the one-way ANOVA followed by multiple comparisons with Bonferroni correction.

Genotoxicity via the comet assay is measured as percent tail DNA obtained by randomly measuring 50 cells per slide from each sample, as recommended by Tice et al. (2000), and obtaining the overall damage $\left({\overline{X}}_{50\kern0.5em \mathrm{cells}}\right)$ value. Values present represent the average of three separate experiments. Oxidative stress was expressed as the difference between the modified and unmodified results as follows:

Oxidative stress (% tail DNA) = FPG-modified (% tail DNA of oxidative stress and background damage) − unmodified (% tail DNA of background damage).

The statistics are presented as one-way ANOVA followed by multiple comparisons with Bonferroni test, *p* < 0.05.

## Results

### Analysis of PAHs in sediments

The results of the sediment analysis revealed 16 PAHs, with the sum of PAHs at the sampling zones increasing as follows: Ilaje < Iddo < Atlas Cove < Apapa (Table 3). Among these, increasing levels of low molecular weight (LMW)-PAHs were found in the order Ilaje < Apapa < Iddo < Atlas Cove, and high molecular weight (HMW)-PAHs in the order Atlas Cove < Iddo < Apapa < Ilaje. The ratio of LMW-PAHs to HMW-PAHs was found to be below 1 in the Ilaje zone, while in other zones it was higher than 1, increasing as follows: Apapa < Iddo < Atlas Cove. In addition, considering the percentage of HMW-carcinogenic PAHs (see Table 3) present in the sediments, Atlas Cove had the lowest level of about 12 %, followed by Iddo at 16 %, Apapa at 28 % and Ilaje at 43 %.

**Table 3:**
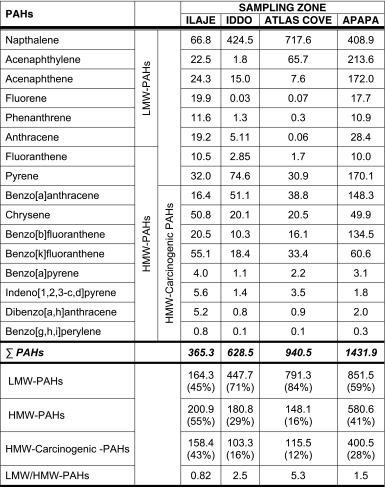
Concentrations of the 16 PAHs extracted from the sediments at the various sampling zones

Values are given in units of micrograms of PAH per kilogram of sediment. The relative concentrations and percentage of total concentrations of low (LMW) and high molecular weight (HMW) and carcinogenic PAHs are given. LMW-PAHs: two- to three-ringed PAHs; naphthalene, acenaphthylene, acenaphthene, fluorene, phenanthrene and anthracene. HMW-PAHs: four- to six-ringed PAHs; fluoranthene, pyrene, benz[a]anthracene, chrysene, benzo[b]fluoranthene, benzo[k]fluoranathene, benzo[a]pyrene, indeno[1,2,3-cd]pyrene, dibenz[a,h]anthracene and benzo[ghi]perylene. HMW-carcinogenic PAHs: benz[a]anthracene, chrysene, benzo[b]fluoranthene, benzo[k]fluoranathene, benzo[a]pyrene, indeno[1,2,3-cd]pyrene, dibenz[a,h]anthracene and benzo[g,h,i]perylene (Classifications based on the IARC monograph (http://monographs.iarc.fr/ENG/Classification/ClassificationsAlphaOrder.pdf and those reported in Chen and Chen 2011)

### Embryotoxicity and teratogenicity

#### Individual PAHs and PAH mixtures

**Fig. 2:**
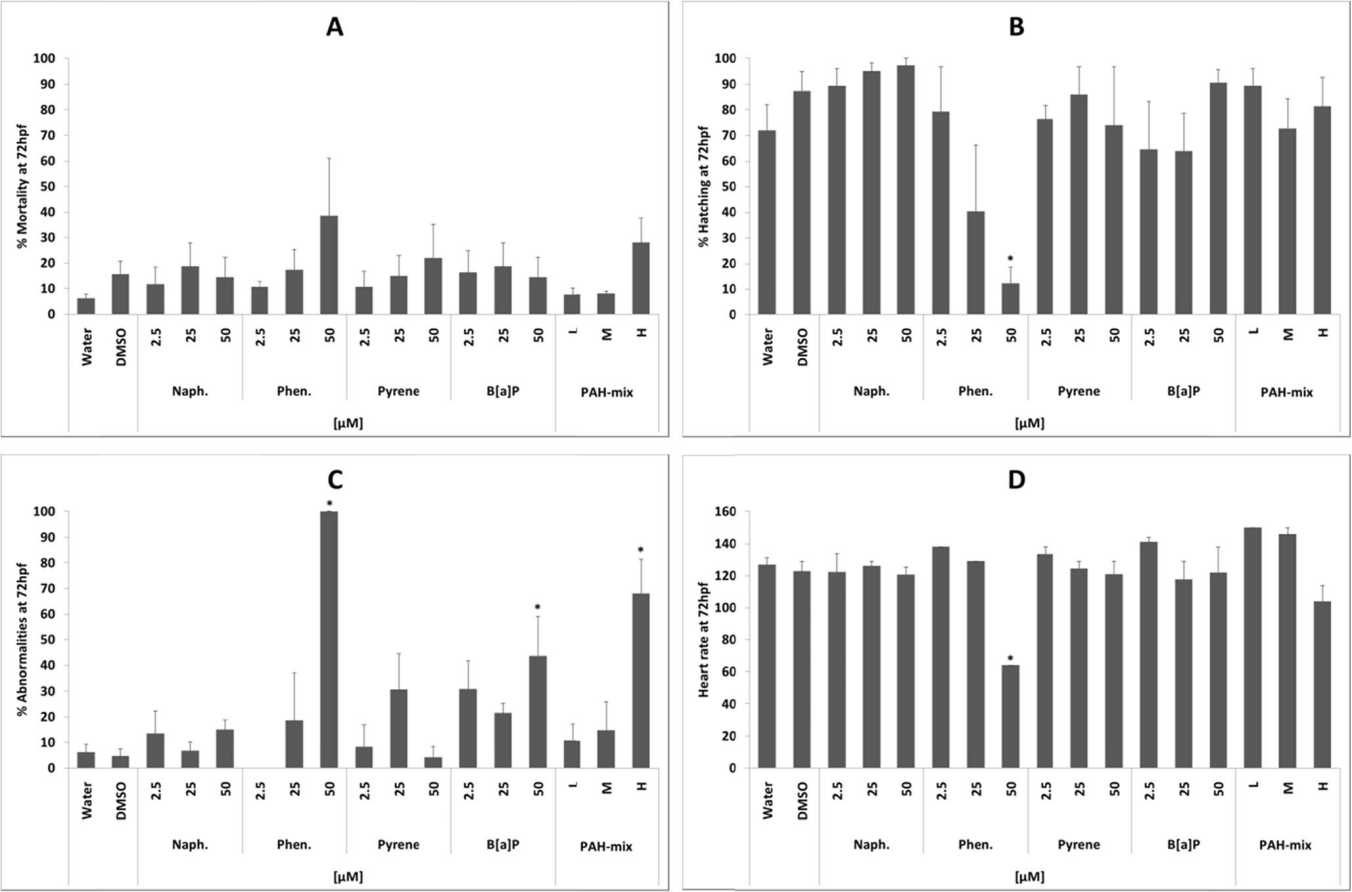
Embryotoxicity and teratogenicity of PAHs to zebrafish. Percent mortality (**a**), hatch rate (**b**), abnormalities (**c**) and heart rate (beats per minute (**d**)) at 72 hpf of exposure to increasing concentration of naphthalene, phenanthrene, pyrene and benzo[a]pyrene and a PAH mixture, as well as DMSO (0.05 % *v*/*v*) and untreated control. The exposure concentrations of individual compounds were 2.5, 25 and 50 μM. The concentrations for the PAH mixtures are set at low (L), medium (M) or high (H); see Table 1 for concentrations of individual PAHs within each mixture. Data are presented as average ± SE, and significant differences (*p* ≤ 0.05) to vehicle control are marked (*). Where error bars are not visible, the number of animals alive or affected between repeats was the same or similar

Phenanthrene proved to be the most potent single PAH tested (Fig. 2a–d), causing a dose-dependent increase in mortality (Fig. 2a), abnormalities (Fig.2c, *p* < 0.05), reduction in hatching (Fig.2b, *p* < 0.05) and depressed heart rate (Fig.2d, *p* < 0.05). The effects of the other PAHs were less dramatic, although 50 μM B[a]P caused a significant (*p* < 0.05) increase in abnormalities (Fig.2c). The PAH mixture showed a significant (*p* < 0.05) increase in abnormalities at the highest concentration (Fig.2c) and a suggestion of elevated levels of mortality (Fig.2a) and depressed heart rate (Fig.2d), but neither of the latter were significantly different from the vehicle control. Developmental abnormalities included stunted growth (Fig. 4b), curvature of the tail (Fig. 4c), delayed hatch (Fig. 4d) and yolk-sac oedema (Fig. 4e, f).

#### Sediment extracts

**Fig. 3:**
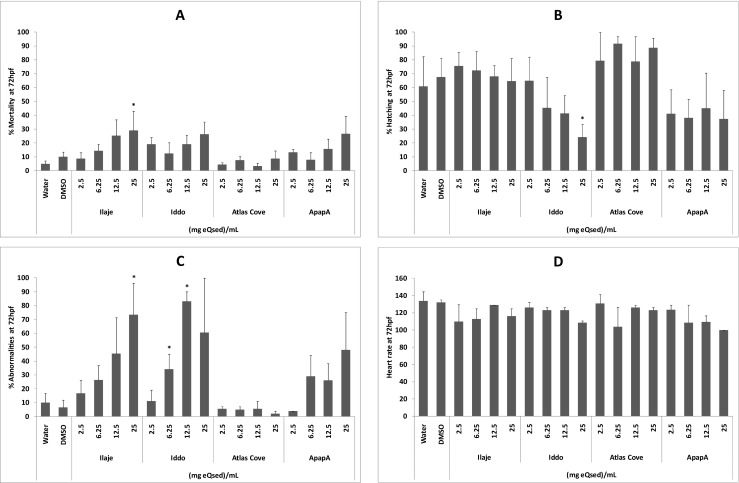
Embryotoxicity and teratogenicity of extracts from Lagos lagoon sediments to zebrafish. Percent mortality (**a**), hatch rate (**b**), abnormalities (**c**) and heart rate (beats per minute (**d**)) at 72 hpf following exposure to increasing concentration of sediment extract (eQsed/mL) from the four sampling sites Ilaje, Iddo, Atlas Cove and Apapa, as well as DMSO (0.25 % *v*/*v*) and untreated control. Data are presented as average ± SE, and significant differences (*p* ≤ 0.05) to vehicle control are marked (*). Where error bars are not visible, the number of animals alive or affected between repeats was the same or similar

**Fig. 4:**
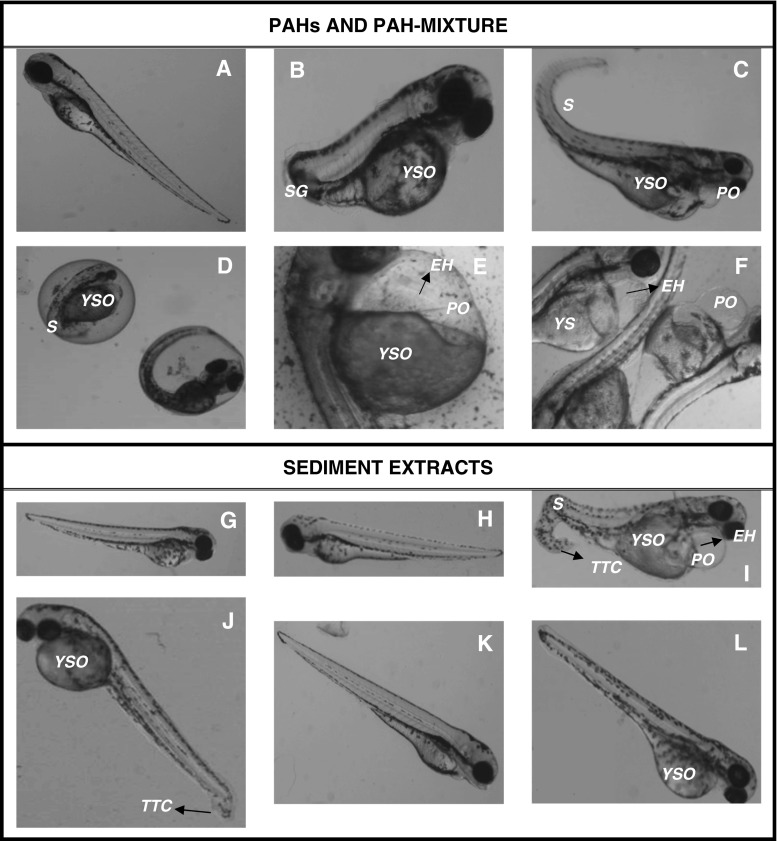
Representative images of teratogenic effects observed at 72 hpf following exposure of zebrafish to individual compounds, PAH mixture and sediment extracts from sampling regions. Exposures were as follows: 0.05 % DMSO control (**a**), 50 μM naphthalene (**b**), 50 μM phenanthrene (**c**), 2.5 μM pyrene (**d**), 25 μM B[a]P (**e**), 50 μM PAH mixture (**f**), water control (**g**), 0.25 % DMSO control (**h**), 25 mg Ilaje eQsed/mL (**i**), 6.25 mg Iddo eQsed/mL (**j**), 25 mg Atlas Cove eQsed/mL (**k**) and 2.5 mg Apapa eQsed/mL (**l**). Examples of defects: *S* scoliosis*, SG* stunted growth*, PO* pericardial oedema*, YSO* yolk-sac oedema*, EH* elongated heart*, TTC* tail tip curvature

The sediment extracts from Ilaje, Iddo and Apapa showed a dose–response increase in mortality, which was only significant (*p* < 0.05) for the Ilaje sediment at the highest concentration (Fig. 3a). Hatching was depressed in embryos exposed to sediments from Iddo and Apapa (Fig. 3b), but significant (*p* < 0.05) only at the highest concentrations of 25 mg eQsed/mL from the Iddo region. Abnormalities were significantly increased in fish exposed to sediments from Ilaje and Iddo (Fig. 3c), whereas no effects were observed for the Atlas Cove sampling zone. None of the four sampling zones gave rise to significantly depressed heart rate (Fig. 3d). Developmental abnormalities included deformed tails, scoliosis, oedemas, haemorrhages and stunted tail development (Table 4, Fig. 4g–l). The most severe and diverse effects came from the Ilaje sediment, whereas no major malformations were observed in the fish exposed to the Atlas Cove sediments.

**Table 4:** Summary of observed teratogenic effects at 72 hpf of selected PAHs, PAH mixture and sediment extracts on zebrafish embryos

Exposure	Teratogenic effects
Naphthalene	• Stunted growth • Yolk-sac oedema
Phenanthrene	• Mild pericardial oedema • Severe yolk-sac oedema • Scoliosis
Pyrene	• Stunted growth • Severe yolk-sac oedema
Benzo[a]pyrene	• Elongated heart • Haemorrhaging
PAH-mixture	• Elongated heart • Haemorrhaging • Severe yolk-sac oedema • Pericardial oedema
Ilaje	• Elongated heart • Haemorrhaging • Severe yolk-sac oedema • Pericardial oedema • Scoliosis • Tail-tip curvature
Iddo	• Yolk-sac oedema • Tail-tip curvature
Atlas Cove	None observed
Apapa	• Haemorrhaging • Moderate yolk-sac oedema

### Genotoxicity results

#### Individual PAHs and PAH mixture

**Fig. 5:**
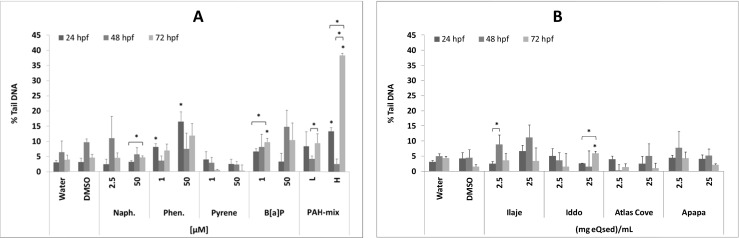
Oxidative damage to DNA in zebrafish measured at 24, 48 and 72 hpf, upon exposure to selected PAHs and PAH mixture (**a**) and sediment extracts (**b**). The exposure concentrations of individual compounds were 2.5, 25 and 50 μM. The concentrations for the PAH mixture are set at low (L) or high (H); see Table 1 for concentrations of individual PAHs within each mixture. Data are presented as average ± SD, and significant differences (*p* ≤ 0.05) in elevated levels are marked (*) in comparison to vehicle control within the same time point, as well as between time points for a given concentration ()

No significant difference in genotoxicity was observed in the unmodified comet assay; consequently, data for the modified comet assay assessing oxidative damage to DNA are presented. Genotoxicity viewed as oxidative stress for the individual compounds showed time- and dose-dependent variations, except for pyrene, which induced the lowest levels of oxidative lesions in DNA (Fig. 5a). Significantly (*p* < 0.05) elevated levels of oxidative damage to DNA compared to the vehicle control at 24 hpf were observed for phenanthrene at both concentrations and for the highest level of PAH mixture (Fig. 5a). No significantly elevated levels were seen for any of the compounds at 48 hpf. At 72 hpf, however, significantly (*p* < 0.05) elevated levels of oxidative stress were seen with 1 μM benzo[a]pyrene and again for the highest level of PAH mixture. Compared over time from 24 to 72 hpf, there was an increase in oxidative stress for naphthalene at 50 μM, for benzo[a]pyrene at 1 μM and for the high PAH mixture. At 48 hpf, significantly increased levels were observed for the PAH mixtures.

### Sediment extracts

All sediment extracts except one, Iddo 25 mg eQsed/mL at 72 h, did not show a significant increase in background or oxidative damage in comparison to the DMSO control at either time points (Fig. 5b). When compared over time, oxidative stress was significantly (*p* < 0.05) increased between 24 and 48 hpf for the 2.5 mg Ilaje eQsed/mL and for the 25 mg Iddo eQsed/mL at 24 and 72 hpf.

## Discussion

The fish embryo acute toxicity (FET) test has been developed as an alternative testing procedure, with an aim to reduce the number of fish used in toxicity testing (Scholz et al. 2008; Lammer et al. 2009), and has been used to evaluate the toxicity of PAHs (Seiler et al. 2014). In the present study, the FET identified HMW carcinogenic PAHs as potential embryotoxic and teratogenic compounds within Lagos lagoon sediment extracts containing a complex mixture of PAHs (Table 3, Figs. 2 and 4). An issue with testing hydrophobic compounds such as PAHs is their propensity to bind to the plastic substrate and/or to be volatilised, altering exposure concentrations. For example, both Vergauwen et al. (2015) and Butler et al. (2013), using a passive dosing system such as PDMS silicone elastomer, reported zebrafish embryo 120 hpf LC50 values of 310 μg/L, and 10 % mortality after 72 h at 423 μg/L phenanthrene, compared with the 40 % mortality at 8900 μg/L (50 μM) observed in the current study (Fig. 2a), suggesting that the current nominal concentrations are an order of magnitude higher than actual exposure concentrations.

Sediment PAH analysis revealed that the highest amount of PAHs was in sediments collected from the Apapa region, followed by decreasing levels of PAHs in sediments from Atlas Cove, Iddo and Ilaje regions (Table 3). An increase in mortality (Fig. 3a) and abnormalities (Fig. 3c), including deformed tails and oedemas (Fig. 4i, j, l), followed a dose–response pattern in embryos subjected to sediment extracts from Ilaje, Iddo and Apapa, but not Atlas Cove. The PAHs present in higher concentrations in these three regions compared to Atlas Cove were acenaphthylene, phenanthrene, anthracene and fluoranthene. Elevated levels of mortality, defects in cardiac function and developmental deformities from treatment with these compounds in particular have been reported previously (Incardona et al. 2004; Butler et al. 2013; Seiler et al. 2014). The highest rate of mortality and abnormalities as well as the lowest rate of hatching and heart rate were observed for phenanthrene with increasing concentrations of the compound and highest dose of 50 μM (Fig. 2a–d). Sublethal effects of phenanthrene have been reported at a concentration of 423 μg/L (~2.4 μM) (Butler et al. 2013). Tail curvature and reduced heart rate have also been observed at a similar nominal concentration of 56 μM phenanthrene (Incardona et al. 2004), which is consistent with the current observations at 50 μM (Table 4, Fig. 4c).

Hatch rate was reduced in embryos exposed to extracts from Iddo and Apapa, both of which contained higher levels of pyrene and benz[a]anthracene compared to the other regions (Table 3, Fig. 3b). Pyrene has been shown to disrupt normal cardiac development and alter expression of defective cardiac differentiation-related genes in zebrafish embryos (Zhang et al. 2012). In the current study, embryos exposed to pyrene displayed a dose-dependent increase in mortality (Fig. 2a), fluctuating hatch rate (Fig. 2b) and abnormalities (Fig. 2c), as well as developmental and teratogenic effects (Table 4, Fig. 4d), but genotoxicity in terms of oxidative stress (Fig. 5a) was negligible. Similarly, benz[a]anthracene has been shown to interfere with development and heart rate in medaka larvae (Le Bihanic et al. 2015), but contrary to our observations, the medaka embryos did not display any decrease in hatch rate. Instead, they had an increase in heart rate with increasing physiological deformities when exposed to benz[a]anthracene-spiked sediments.

Although four compounds were found in higher levels in the three regions displaying dose-dependent embryotoxicity, and two compounds found in excess in sediments giving rise to lowered hatch rate, it is not possible to say that these are responsible for the increase in the specific types of physiological changes observed. It is known that different compounds in various combinations may alter activating/metabolising enzymes in biological systems whereby additive, synergistic or even diminishing effects are achieved. These various effects are clearly demonstrated by the fluctuating, rather than consistent, levels of damage from the PAH mixture, where mortality and teratogenic endpoints (Figs. 2a–d and 4f) show a moderate effect, whereas oxidative stress (Fig. 5a) and abnormalities (Fig. 2c) become highly significant over time. These observations also signify that it is not solely the sum of pollutants present in the sediments that determines its toxicity. In fact, Atlas Cove, which contains the second highest level of PAHs at 940.5 μg/kg sediment in the extracts, had the smallest effect on the developing embryos (Figs. 2a–d and 4k). This is consistent with the composition of the extract, which consists of high levels of LMW-PAHs (791.3 μg/kg—84 %), low levels of HMW-PAHs (148.1 μg/kg—16 %) and the lowest level of carcinogenic compounds (115.5 μg/kg—12 %) (Table 3). Generally stated, carcinogenic PAHs are more common among four to six ring structures (Pott and Heinrich 1990), which in these sampling zones are highest at Ilaje, followed by Apapa and Iddo. However, the highest level of toxicity from a single compound in our tests is attributed to that of a three-ring structure, phenanthrene, yet the most toxic sediments were those containing high levels of HMW carcinogenic PAHs. This further supports the contributions from various compounds in complex mixtures, giving rise to diverse physiological effects, which makes it difficult to evaluate and extrapolate overall toxicity by observing single components. In addition, potential PAH carcinogenicity is based on mammalian data, hence species-specific physiological factors also play a key role in compound toxicity. But the fact that extracts containing HMW-PAHs that are known or suspected mammalian carcinogens have extensive effects on the embryos is a cause for concern and warrants further investigation. This draws similarities to expected/observed toxic effects of HMW-PAHs in mammals and would indicate that the zebrafish embryo test is a potential model for assessing the toxicity of complex PAH mixtures.

There was generally a low level of genotoxicity (Fig. 5a, b), and thus, it would appear that DNA damage could not be linked to developmental abnormalities attributed to oxidative stress, perhaps with the exception of the high PAH mixture after 72 hpf, which gave rise to an eightfold increase in oxidative lesions (Fig. 5a) compared to the vehicle control. Although the PAH mixture is “mimicking” a complex mixture, elevated levels of oxidative stress by the extracts are only fourfold higher at most. Significant increases in oxidative stress were observed only for the Ilaje and Iddo extracts, but the overall low levels suggest that the developmental changes/abnormalities observed are probably not due to these types of genotoxic damages. It is worth noting that DNA damage repair as a response to oxidative stress is often a rapid process, and other biomarkers of oxidative damage, such as lipid peroxidation, would possibly be more appropriate for measurement during long-term exposures.

## Conclusion

This study showed that the Lagos lagoon sediments containing complex mixtures of pollutants, particularly the Ilaje sediment, have embryotoxic and genotoxic properties, which correlate to HMW-PAHs of the sort that are known or are suspected mammalian carcinogens. This highlights the importance of (1) considering pollutant composition in complex mixtures, not just absolute concentrations of pollutants, (2) assessing complex mixtures rather than single compounds to obtain more realistic results on harmful effects and (3) the pertinence of using zebrafish model system in evaluating complex mixtures that may pose a serious risk to human and environmental health. Lastly, due to the demonstrated embryotoxic and genotoxic properties of the sediments, it is recommended that envirovigilance and regulation of activities at the stations/zones of concern be considered.
